# 

**Published:** 1902-10-18

**Authors:** 


### NOTICE TO CORRESPONDENTS.

**All MSS., Letters, Books for Review, and other matters intended for the Editor should be addressed The Editor. "The Hospital," The Hospital Building, 28 & 29 Southampton Street, Strand, W.C. The Editor cannot undertake to return rejected MS., even when accompanied by stamped directed envelope.**

**Notice to Advertisers and Subscribers.**

**All Advertisements, Orders for Copies of The Hospital, and Business Communications should be addressed to THE MANAGER, (Not to the Editor), The Hospital, 28 & 29 Southampton Street, Strand, London, W.C.**

The Cost of Subscription, post free, is as follows.—Per week, 2½d.; per quarter, 2s. 8d.; per half-year, 5s. 3d.; per annum, 10s. 6d. Foreign:—Per annum, 16s. 2d., payable, in all cases, in advance.

**NOTICE.—Vol. XXXII. of "The Hospital" (April to September 1902) in handsome cloth binding will shortly be on sale at the office of this Journal, price, 7s. 6d. Cases for binding the Numbers contained in this Volume 1s. 6d. Index to Vol. XXXII., 2½d., post free.**

**The Monthly Part for October is now ready. Price 6d., by post 8d.**

### THE STUDENT OF MEDICINE.

### APPOINTMENTS.

**J. Hill Abraham**, M.D.Lond., M.R.C.P., appointed Honorary Physician to the Royal Infirmary, Liverpool. **J. Beatty**, M.D.Dub., D.P.H., R.C.P.S.I., appointed Medical Officer of Health for the Borough of Northampton. **Walter Briggs**, M.B., Ch.B.Vic., appointed Junior House-Surgeon of the Blackburn and East Lancashire Infirmary. **Frederick H. Flack**, M.B., Ch.B.Vict., appointed Senior House-Surgeon of the Blackburn and East Lancashire Infirmary. **Andrew Fullerton**, M.D., B.Ch., F.R.C.S.Irel., appointed Honorary Assistant Surgeon to the Royal Victoria Hospital, Belfast. **Charles W. Gilbert**, M.R.C.S., L.R.C.P.Lond., appointed District Medical Officer of the Orsett Union. **L. P. Gordon**, M.R.C.S., L.R.C.P.Lond., appointed District Medical Officer of the Ashbourn Union. **Charles John Harnett**, M.D.Lond., M.R.C.S., L.R.C.P., appointed Surgeon and Agent to the Admiralty at Margate. **James Holmes**, M.D., appointed Certifying Factory Surgeon for the Bury District of the County of Lancaster. **W. M. Jones**, L.R.C.P., L.R.C.S.I., appointed Resident Assistant Medical Officer of the Liverpool Parish Workhouse. **D. L. Lindsay**, L.R.C.P., L.R.C.S.Edin., appointed Assistant Medical Officer of the Toxteth Park Workhouse. **A. Lyth**, M.R.C.S., L.R.C.P., appointed Assistant House-Surgeon to the Rotherham Hospital and Dispensary. **C. A. Morgan**, M.R.C.S.Eng., appointed District Medical Officer of the Dorchester Union. **James Neal**, L.R.C.P.Lond., M.R.C.S., appointed Medical Officer of the Royal Small Arms Establishments at Birmingham. **Laurence Potts**, M.R.C.S.Eng., L.S.A., appointed Medical Officer to the School for the Blind, Leatherhead. **John Powell**, L.R.C.P. and L.R.C.S., appointed Admiralty Surgeon and Agent for the Barry (South Wales) District. **B. E. Sansom**, L.S.A., appointed Assistant Medical Officer of the Camberwell Parish Workhouse. **E. W. Sharp**, M.B., Ch.B., appointed Resident Assistant Medical Officer of the Halifax Union Workhouse. **Edward Sharpley**, M.R.C.S., L.R.C.P., appointed Medical Officer for the Yarborough District of the Louth Union. **James F. Smith**, M.B., C.M.Edin., appointed Certifying Factory Surgeon for the Withorn District of the County of Wigtown. **E. J. Walls**, M.R.C.S., L.R.C.P.Lond., appointed Certifying Factory Surgeon for the Halesowen District of the County of Worcester. **H. G. Weston**, M.R.C.S., L.R.C.P.Lond., appointed Assistant Medical Superintendent to the Lewisham Infirmary. **D. S. Wylie**, M.B., Ch.B.Vict., has been appointed House-Surgeon to St. Peter's Hospital for Stone, Henrietta Street, Covent Garden.

### "The Hospital" Institutional Library.

"Hospitals and Asylums of the World." 4 Vols., with a Portfolio of Plans, complete £12 12s.

"Burdett's Hospitals and Charities, 1902." 5s.

"Cottage Hospitals, General, Fever, and Convalescent." 10s. 6d.

"Hospital Expenditure: The Commissariat." 2s. 6d.

"A Legal Handbook for the Use of Hospital Authorities." 2s. 6d.

"The Cottage Hospital Case Book or Register of Patients." 10s. and 12s. 6d.

"The Furnishing and Appliances of a Cottage Hospital." 6d.

All these are published by the Scientific Press, Ltd., and may be obtained through any bookseller, or direct from the publishers, 28 and 29 Southampton Street, Strand, London, W.C.

